# Healing With the Arts: Older Adult Recovery Narratives

**DOI:** 10.1093/geroni/igaa057.2347

**Published:** 2020-12-16

**Authors:** Karen Fortuna, Jessica Brooks

**Affiliations:** 1 Dartmouth College, Concord, New Hampshire, United States; 2 Columbia University, New York, New York, United States

## Abstract

Recovery narratives are an effective healing art medium that support individual development by virtue of the shared human experience transmitted through each story. Older adult recovery narratives are unique as they share their experiences of aging with a mental health conditions to support others with similar difficulties. Older adult recovery narratives offer encouragement of self-determination in late life, share lived experience of aging with a mental health condition, and promote age-related self-management skills development. The RecoverYdia smartphone app provides an online community for older adults with a lived experience of a mental health condition. RecoverYdia subscribers can search through hundreds of relevant videos and find the storyteller who tells the viewer’s story, prompts them to reach out for help, and eventually inspires them to help others. This presentation will discuss the state of evidence regarding the evidence for recovery narratives across the globe and offer a RecoverYdia technology demonstration.

